# Effects of short-term betaine supplementation on muscle endurance and indices of endocrine function following acute high-intensity resistance exercise in young athletes

**DOI:** 10.1080/15502783.2022.2041988

**Published:** 2022-03-22

**Authors:** Hamid Arazi, Shima Aboutalebi, Behzad Taati, Jason M. Cholewa, Darren G. Candow

**Affiliations:** a Department of Exercise Physiology, Faculty of Sport Sciences, University of Guilan, Rasht Iran; b Department of Exercise Physiology, College of Health Sciences, University of Lynchburg, Lynchburg, VA USA; c Faculty of Kinesiology and Health Studies, University of Regina, Regina, SK, Canada

**Keywords:** Testosterone, cortisol, lactate, anabolic

## Abstract

**Objective:**

This study examined the effects of short-term betaine supplementation on muscle endurance, plasma lactate, testosterone and cortisol levels, and the testosterone to cortisol (T/C) ratio in response to acute resistance exercise (RE).

**Method:**

Using a double-blind, crossover study design, 10 handball players (age ± SD = 16 ± 1 yrs) without prior-structured RE experience performed a high-intensity RE session (leg press followed by bench press; 5 sets to volitional fatigue using 80% baseline 1 repetition maximum (1RM)), before and after 14 days of either placebo (maltodextrin) or betaine (2.5 g·d^−1^) supplementation. A 30-day washout period separated each treatment. 48 h prior to testing sessions, participants recorded their food intake and did not perform strenuous exercise. Venous blood was sampled before supplementation, and before and after each RE session.

**Results:**

After betaine supplementation, participants performed more repetitions (*p* < 0.001) during the leg press (Betaine: 35.8 ± 4.3; Placebo: 24.8 ± 3.6, Cohen’s *d* = 2.77) and bench press (Betaine: 36.3 ± 2.6; Placebo: 26.1 ± 3.5, Cohen’s *d* = 3.34). Betaine resulted in lower post-exercise cortisol (Betaine: 7.6 ± 1.7; Placebo: 13 ± 3.4 µg.dL^−1^, *p* = 0.003, generalized eta squared ($\eta_{G}^{2}$) = 0.49) and lactate (Betaine: 5.2 ± 0.3; Placebo: 6 ± 0.3 mmol.L^−1^, *p* < 0.001, $\eta_{G}^{2}$ = 0.96) and higher total testosterone (Betaine: 15.2 ± 2.2; Placebo: 8.7 ± 1.7 ng.mL^−1^, *p* < 0.001, $\eta_{G}^{2}$ = 0.87) and T/C ratio (Betaine: 0.21 ± 0.05; Placebo: 0.07 ± 0.02, *p* < 0.001, = 0.82).

**Conclusions:**

Two weeks of betaine supplementation improved upper- and lower-body muscle endurance and influenced indices of endocrine function following an acute session of high-intensity RE in adolescent handball players.

## 1. Introduction

Nowadays, the use of sports supplements with the aim of improving performance and recovery seems to be common among athletes of various sports [1]. Betaine or trimethylglycine was first isolated as a naturally occurring byproduct of sugar beet refinement [2,3]. Betaine may also be consumed in other food sources such as wheat germ, wheat bran, spinach, and wheat bread and also from oxidation of dietary choline in the liver and kidneys [4,5]. Betaine serves three major physiological roles, including an important organic osmolyte, a methyl group donor, and a possible protective agent against intracellular protein denaturation under stressful conditions [2,6]. Betaine acts as an osmolyte to sustain cell hydration and function in response to a variety of physiological stressors. Additionally, betaine serves proteins as a possible chaperone to maintain native folded conformation and stability without disrupting other cellular processes [2]. Finally, betaine has been suggested to increase anaerobic work and reduce perceived exertion and fatigue by increasing free choline, which in turn may lead to an increase of acetylcholine synthesis in motor neurons [7]. These important properties may help to maintain a more favorable environment for excitation-contraction coupling during intense exercise. Given the potential physiological properties, it has been proposed that supplementation with betaine may provide ergogenic effects in humans [8].

he first use of betaine as an ergogenic aid was reported by Borsook et al. [9] whereby chronic supplementation at ~14 mg/kg of body mass enhanced general strength and endurance in patients with poliomyelitis. In a recent study, Nobari et al. [10] evaluated the chronic effects of betaine supplementation in professional youth soccer players and concluded that 14 weeks of supplementation (2 g/day) increased testosterone and testosterone:cortisol (T/C) ratio during a competitive season. The efficacy of betaine to enhance the quality of a resistance exercise (RE) session was first studied by Maresh et al. [11], where ingestion of a betaine supplement for 14 days significantly improved bench press throw and vertical jump power, isometric bench press force, and isometric squat force in recreationally trained men. Subsequently, other investigators examined performance benefits from sub-chronic (2 weeks) betaine supplementation in RE. The results of these studies provide equivocal evidence, with some reporting improvements in strength and power [12, 13], and others reporting no improvements in these performance parameters [8,14,15]. Nevertheless, it has been suggested that betaine may have a potential ergogenic effect in RE protocols with high metabolic stress [2]. For example, Hoffman et al. [8] showed that 15 days of betaine supplementation (2.5 g/day) improved squat exercise repetitions to fatigue with 75% of 1RM, without similar improvement in the bench press performance. The authors stated that the larger muscle mass involved in the leg press exercise compared with the bench press may have been affected to a greater extent from betaine supplementation. In this trial, participants also completed more repetitions at or above 90% power output. In support of these results, reductions in muscle fatigue [16] and increases in bench press total volume (~6.5%) and repetitions to fatigue [15] have been reported after a sub-chronic period (i.e. 14–15 days) of betaine ingestion. On the other hand, Lee et al. [13] reported no differences in total repetitions during 3 sets of 85% 1RM bench press and back squat with 2 weeks of betaine supplementation.

To date, the small body of research involving betaine and RE has focused primarily on performance factors (i.e. strength, power, endurance) [8, 14, 16, 17, 13], with very little attention given to indices of endocrine function [6,12,15]. Furthermore, the effect of betaine and RE in adolescent athletes is unknown. Preliminary research has shown that betaine supplementation at 2.5 to 3 g/day for 14 days in trained men led to a non-significant change in blood lactate [15], and a significant increase in growth hormone (GH) and IGF-1 [6,12] in response to acute bouts of RE. Although these findings suggest a positive effect of short-term betaine supplementation on the endocrine response to exercise and lactate (as a proxy of anaerobic energy production), more research is needed to substantiate the safety and ergogenic potential of this nutrient in adolescent athletes. Additionally, current data concerning the potential effects of betaine on muscle endurance is conflicting and requires additional research [8]. Therefore, the purpose of this study was to investigate the effects of betaine supplementation on muscle endurance, blood lactate, testosterone and cortisol levels, and T/C ratio in response to an acute bout of RE in adolescent athletes.

## 2. Materials and methods

### Participants

After approving the present research protocol by the Institutional Review Board of the University (DT-14925), ten adolescent male handball players (mean age ± SD: 16 ± 1 yrs; height, 182 ± 7.3 cm; body mass, 78.8 ± 11.4 kg; body mass index, 23 ± 3.3 kg/m^2^; ≥3 years of handball training experience) who performed at least three handball training sessions per week participated in this study. Following an explanation of the purpose, the experimental procedures, possible risks and benefits, written informed consent was obtained from all participants and their parent or legal guardian before beginning the study. All participants were healthy, with no physical injury or major chronic diseases, such as cardiovascular disease, or diabetes based on a Confidential Medical and Activity Questionnaire. Furthermore, although all participants had prior non-structured RE experience, they were not involved in regular RE for at least 3 months prior to study enrollment.

**Figure 1.**
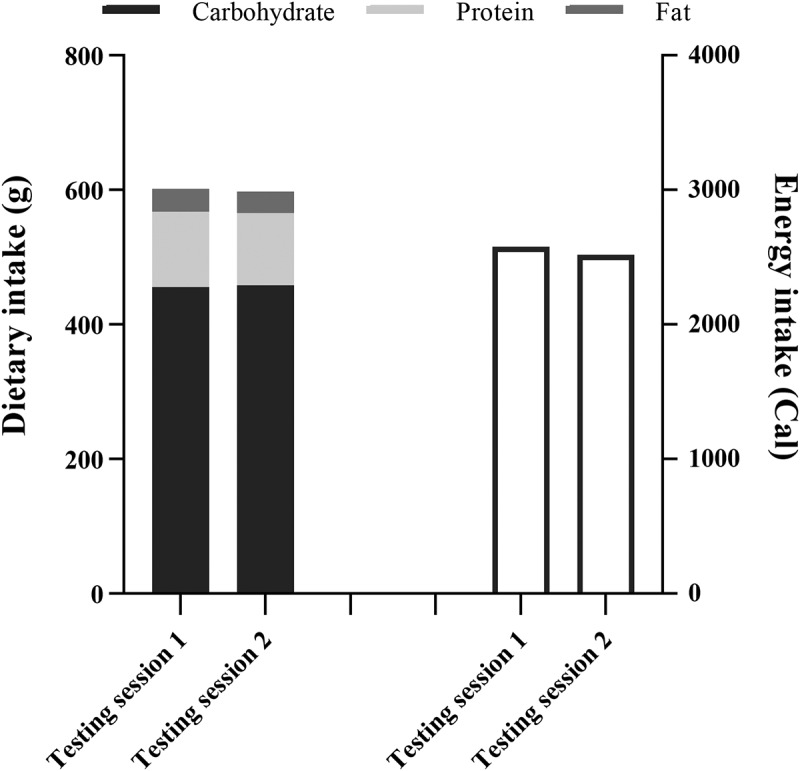
Mean of dietary intake 48 h before either testing session.

Participants maintained their usual handball training routine during both placebo and betaine phases but were asked to refrain from strenuous physical activity throughout the 48 h period prior to each testing day. Participants had not consumed dietary supplements containing creatine monohydrate or drugs/medications known to increase performance for at least a year before the start of the study and were instructed not to consume any additional nutritional supplements during the study. In addition, participants were instructed to maintain their habitual diets throughout the study. To further control habitual dietary intake, participants received written and verbal instructions to record the type and portion sizes of daily foods consumed 48 h before the first testing session. Additionally, to maintain consistency, they were asked to mimic this diet 48 h before the second testing session. Relevant nutritional data are presented in Figure 1. There were no statistically significant differences in any of the dietary measures before the first and second testing occasions.

### Study procedures

**Figure 2.**
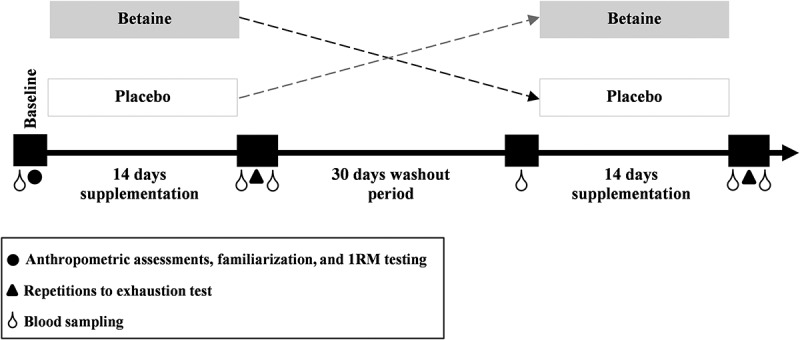
Research time line. 1RM, 1 repetition maximum.

The study used a randomized, placebo-controlled, double-blind, crossover design involving two 14-day periods involving betaine and placebo ingestion, separated by a 30-day washout period. All procedures, including data collection, familiarization and testing sessions, were time-matched between visits and were completed between 8:00 and 11:00 AM in a temperature-controlled lab (22–24 °C) to minimize potential diurnal variations in hormones and RE performance. After an overnight fast of at least 8 h, all participants reported to the laboratory for their initial screening visit and initial blood samples were drawn following 30 min of rest on a comfortable chair. During this visit, anthropometric assessments were obtained and the participants familiarized themselves with RE equipment (under supervision), including correct lifting technique, range of motion and suitable breathing for both exercises used in the RE protocol (leg press and bench press). They then performed a 1 repetition maximum (1RM) test to determine their maximal strength and were subsequently given their supplement or placebo at the end of this session in a randomized, double-blind manner. Each supplementation period involved 14 days of betaine or placebo intake. Immediately following each intervention (betaine and placebo), participants returned to the laboratory in a fasted state to perform the RE performance test (repetitions to exhaustion). Blood samples were also collected before and following the test. Figure 2 shows the study protocol timeline.

### Anthropometric measurements

At the beginning of the first visit, body weight (SECA 803, Hamburg, Germany), height (SECA 206, Hamburg, Germany) and body mass index (BMI) were measured. A calibrated caliper (1127A, Lafayette Instruments, Indiana, USA) was also used to measure skinfolds thickness at three sites including chest, abdomen and thigh on the right side of the body. Each site was measured three times using the sequence mentioned above. Body fat percentage was then calculated using the equations provided by Jackson and Pollock [18], and Siri [19].

### Supplementation procedures

Each participant consumed either 1.25 g of betaine (Betaine Anhydrous Trimethylglycine Powder, Bulk Supplements Inc., NV, USA) or placebo (maltodextrin) mixed in 125 mL of warm water (for better dissolution) twice per day (morning and evening, 60 min after a meal; total amount of 2.5 g/day) for 14 days. Betaine powder and maltodextrin were identical in appearance and taste and were not distinguishable by the participants or investigators. Participants received powder packets (similar in shape and size) containing either the betaine or placebo that were coded by someone not involved in the study. Therefore, neither the investigators nor the participants were aware of the contents until completion of statistical analyses. Participants were provided with bottles of water and were instructed to mix the contents of each packet in 125 ml of warm water and to consume this solution immediately after preparation. To ensure supplementation compliance, a researcher called the participants every night and the empty packets were also collected on a weekly basis.

### Maximal strength testing

To assess upper- and lower-body strength, a 1RM test was performed on the bench press and leg press exercises, respectively. All participants visited the laboratory before the supplementation period. Ten minutes of pedaling a cycle ergometer was followed by lower- and upper-limb stretching exercises and two warm-up sets of leg press and bench press with no load prior to 1RM testing. Each participant’s 1RM was determined using standardized methods described by the National Strength and Conditioning Association [20]. In brief, participants performed a specific warm-up set of the given exercise with five repetitions carried out at ∼50% of participant’s perceived 1RM followed by 1 to 2 sets of 2–3 repetitions at a load corresponding to ∼60–80% 1RM. Participants then performed sets of one repetition of increasing load for 1RM determination. A rest period of at least 3 min was provided between each set to avoid fatigue. All 1RM determinations were made within four to five trials.

Standard techniques were used for both strength tests as follows: the bench press was performed by lying flat on a bench, with feet flat on the ground and buttocks and shoulders touching the bench. The bar was grasped at slightly wider than shoulder width apart and the elbows were at right angles at the lowest point. The test started with the arms fully extended, holding the weight directly above the chest. The weight was lowered at a controlled speed and with a smooth motion, to just touch the chest and then returned to the starting position. The participants given support for ‘lift off’ during the bench press, but they required to un-rack and rack themselves. Moreover, the spotters were ready to help them during the performance if needed. The leg press was performed by a standard 45-degree machine. Each player sat down on the machine and placed the legs on the footplate at about hip-width apart while ensuring that the heels were flat. In this position, the legs formed an angle of about 90 degrees at the knees. After starting, the footplate was pressed until the legs were fully extended, without locking the knees. The footplate was then pushed with the heels and returned to the starting position.

### Repetitions to exhaustion

After a warm-up period that was similar to what the participants did in the 1RM test session, they completed five sets to exhaustion on both the leg press and bench press exercises at 80% of their previously determined 1RM, with 2 min rest intervals between the sets. Furthermore, the leg press testing was separated from the bench press testing by a 5 min passive rest. Participants were encouraged to perform as many repetitions as possible using the abovementioned lifting techniques and the total number of proper repetitions performed was recorded.

### Blood sampling and analyses

At the beginning of each 14-day supplementation period, and before and after each testing occasion, 5 mL of fasting blood were drawn from a superficial antecubital vein via a needle. Blood samples were collected into ethylenediaminetetraacetic acid (EDTA) containing tubes and centrifuged at 1000 rpm for 20 min at 4 °C. Obtained plasma was aliquoted and stored at −20 °C until later analyses. Commercially available human ELISA kits were used in duplicate to measure plasma total testosterone (sensitivity: 0.07 ng/mL, RE52151, IBL International, Germany), and cortisol concentrations (sensitivity: 0.24 µg/dL, RE52061, IBL International, Germany) according to the procedures provided by the manufacturer. The coefficient of variation for each assay was 3.1% for testosterone, and 2.1% for cortisol. Plasma samples were also analyzed for lactate concentration using a colorimetric assay kit according to instructions of the manufacturer (sensitivity: 0.01 mg/dL, Greiner Diagnostic, Germany). In brief, all reagents were prepared and mixed thoroughly before use. 50 µL of plasma sample and 50 µL of Reaction Mix were added into the wells of microtiter plate. The well contents then were mixed thoroughly and incubated for 30 min at 37°C protected from light. Thereafter, the plate was read with a spectrophotometric microplate reader at 540 nm. All measurements were performed on the same day and reported in respective SI units.

### Statistical analyses

All data were analyzed using the SPSS software (v. 18®, Inc. Chicago, IL) for the Windows and statistical significance was set at an alpha level of *p* ≤ 0.05 for all tests. Data were first tested for normality using the Shapiro–Wilk test. Blood variables (i.e. testosterone, cortisol, T/C ratio, and lactate) were analyzed using repeated measures ANOVA with two conditions (betaine or placebo) × 3 times (within-subject factors for blood measures). A 2 condition × 5 times (sets of repetitions to exhaustion) ANOVA was used to analyze bench press and leg press. The sphericity assumption (Mauchly’s test) was met and Bonferroni post hoc test was used to determine specific pairwise differences when appropriate. Nevertheless, due to non-normal distribution, muscle endurance data were analyzed using the Friedman test. Further, Dunn’s pairwise post hoc tests were conducted with a Bonferroni correction applied to find out which pairs were different.

In order to correct the bias typically found with small samples and using customized excel spreadsheets (Excel 2010, Microsoft, Redmond, USA), generalized eta squared ($\eta_{G}^{2}$) [21] and Kendall’s W [22] were calculated to express effect size (ES). The ES statistics were interpreted as small ($\eta_{G}^{2}$ ≤ 0.02; 0.1 ≤ W < 0.3), medium (0.02 < $\eta_{G}^{2}$ < 0.13; 0.3 ≤ W < 0.5), large (0.13 < $\eta_{G}^{2}$ ≤ 0.26; 0.5 ≤ W), and very large ($\eta_{G}^{2}$ > 0.26) [23]. Cohen’s *d* was also calculated for total repetitions in the upper- and lower-body workout and was considered as follows: trivial (<0.2), small (0.2 ≤ *d* < 0.5), medium (0.5 ≤ *d* < 0.8) and large (0.8 ≤) [24].

## 3. Results

The supplement and testing sessions were well tolerated by all athletes, with no adverse events reported. Supplementation compliance was 100%. 1RM records (mean ± SD) for the leg press and bench press were 291.3 ± 26.3 and 52 ± 7.7 kg, respectively.

### Upper- and lower-body muscle endurance

**Figure 3.**
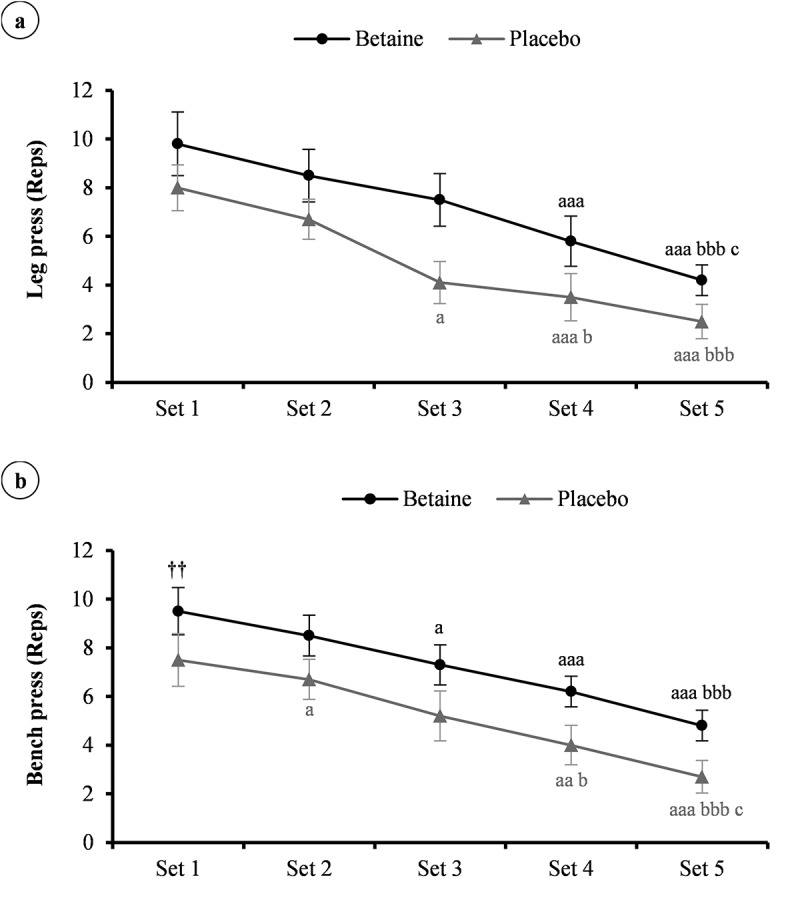
The number of repetitions (reps) per set of the leg press (a), and bench press (b) workout in adolescent male handballers (n = 10) after 14 days betaine supplementation vs. placebo intake. ^a^ *p* < 0.05, ^aa^ *p* < 0.01, and ^aaa^ *p* < 0.001 vs. set 1; ^b^ *p* < 0.05, and ^bbb^ *p* < 0.001 vs. set 2; ^c^ *p* < 0.05 vs. set 3.

Considering the Friedman test, significant differences were noted between the conditions for the leg press (X^2^ = 85.5, *p* < 0.001, W = 0.95) and bench press (X^2^ = 84.6, *p* < 0.001, W = 0.94) repetitions over 5 sets to exhaustion with 80% of 1RM. In confirmation, paired sample t-test revealed that total repetitions in the leg press (35.8 ± 4.3 vs. 24.8 ± 3.6 reps, Cohen’s *d* = 2.77, 95% CI = 1.36 to 4.16) and bench press (36.3 ± 2.6 vs. 26.1 ± 3.5 reps, Cohen’s *d* = 3.34, 95% CI = 1.69 to 4.97) exercise were greater after betaine supplementation (*p* < 0.001). The subjects performed more repetitions during each set of the leg press (Figure 3(a); set 1, *p* = 0.99; set 2, *p* = 0.99; set 3, *p* = 0.16; set 4, *p* = 0.99; set 5, *p* = 0.1) and bench press (Figure 3(b); set 1, *p* = 0.99; set 2, *p* = 0.99; set 3, *p* = 0.73; set 4, *p* = 0.9; set 5, *p* = 0.99) exercises after betaine treatment, but there were no statistically significant interactions.

### Resting levels of blood variables

**Figure 4.**
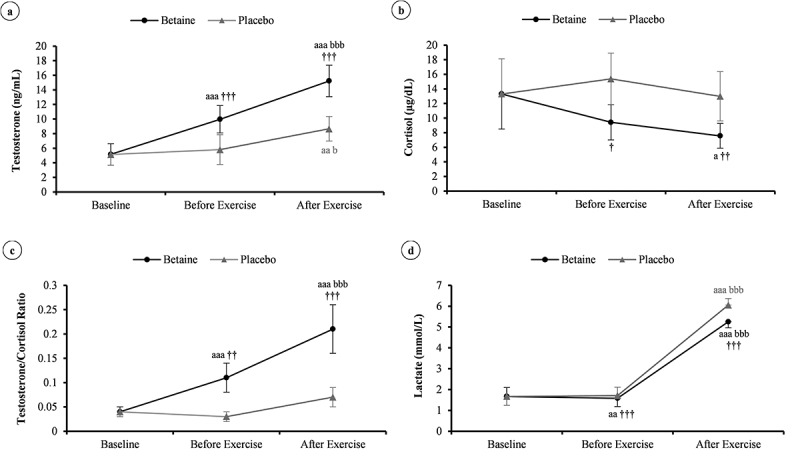
Changes in plasma testosterone (a), cortisol (b), testosterone to cortisol ratio (c), and lactate (d) in response to a session of high-intensity resistance exercise in adolescent male handballers (n = 10) after 14 days betaine supplementation vs. placebo intake. ^a^ *p* < 0.05, ^aa^ *p* < 0.01, and ^aaa^ *p* < 0.001 vs. baseline; ^bbb^ *p* < 0.001 vs. before exercise; ^†^ *p* < 0.05, ^††^ *p* < 0.01, and ^†††^ *p* < 0.001 vs. placebo.

The effects of short-term betaine intake on the blood variables were evaluated by comparing the before-exercise data between the betaine and placebo conditions. There was no significant difference between the conditions at baseline (*p* = 0.99). After betaine supplementation, plasma total testosterone (from 5.15 ± 1.5 to 10 ± 1.9 ng.mL^−1^) and T/C ratio (0.04 ± 0.01 to 0.11 ± 0.03) were significantly increased compared to baseline concentrations (*p* ≤ 0.001) and placebo condition (*p* ≤ 0.01), whereas resting cortisol (9.4 ± 2.4 vs. 15.4 ± 3.5 µg.dL^−1^; *p* = 0.02) and lactate (1.6 ± 0.4 vs. 1.7 ± 0.4 mmol.L^−1^; *p* < 0.001) were decreased, as presented in Figure 4(a) to -d.

### Responses after exercise

**Figure 5.**
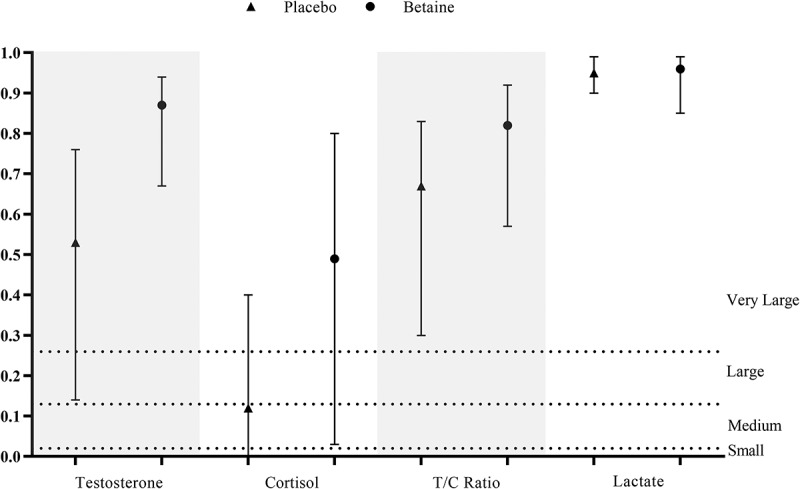
Effect size estimates (generalized eta squared) after 14 days betaine supplementation vs. placebo intake for each blood parameter measured in this study.

The responses of blood variables following a single session of RE were compared between the betaine and placebo conditions to determine the effect of a 2-week betaine administration (Figure 4(a-d)). The ES statistics (i.e. $\eta_{G}^{2}$) with the 95% CI are also shown in Figure 5. Whereas post-exercise cortisol (7.6 ± 1.7 vs. 13 ± 3.4 µg.dL^−1^, *p* = 0.003, $\eta_{G}^{2}$ = 0.49) and lactate (5.2 ± 0.3 vs. 6 ± 0.3 mmol.L^−1^, *p* < 0.001, $\eta_{G}^{2}$ = 0.96) were lower in the betaine condition, plasma total testosterone (15.2 ± 2.2 vs. 8.7 ± 1.7 ng.mL^−1^, *p* < 0.001, $\eta_{G}^{2}$ = 0.87) and T/C ratio (0.21 ± 0.05 vs. 0.07 ± 0.02, *p* < 0.001, $\eta_{G}^{2}$ = 0.82) were significantly higher than those of placebo.

## 4. Discussion

The first aim of this study was to examine if short-term (i.e. 14 days) betaine supplementation influenced upper- and lower-body muscle endurance in adolescent athletes. The acute responses of lactate, testosterone and cortisol following the high-intensity RE session were also measured before and after the supplementation period. Betaine supplementation enabled participants to perform more repetitions during the bench press and leg press and also reduced post-exercise lactate concentration. We also observed that betaine amplified the testosterone responses and dampened the cortisol response to a single session of RE compared to placebo. Moreover, in comparison to baseline values, betaine supplementation led to an increase in resting testosterone and the T/C ratio.

Conflicting results have been reported related to the effect of chronic (2 to 10 weeks) betaine supplementation on RE performance [2]. Although Lee et al. reported improvements in upper- and lower-body absolute strength and power [13], most studies have failed to replicate these results when measuring strength via 1RM or isokinetic dynamometry [3]. On the other hand, several studies have reported an ergogenic effect of 2.5 g/day betaine supplementation on muscular endurance outcomes [2]. Studies that have reported an ergogenic effect have typically employed multiple sets with moderate (60–80% 1 RM) intensities [8,15,16]. The results of this investigation lend support to the hypothesis that betaine supplementation may enhance RE protocols that challenge muscular endurance with greater metabolic demands [25].

A seemingly paradoxical finding in this study was the relationship between total repetitions and plasma lactate. Significant correlations between anaerobic power output and plasma lactate have been reported in multiple studies [26,27]; however, despite performing significantly more work, post-exercise plasma lactate was significantly less with betaine supplementation in the present study. Similar to our results, Trepanowski et al. [15] reported significantly greater repetitions (6.5%) and a non-significant trend for lower post-exercise lactate following 10 sets of bench press to failure with betaine supplementation, and Apicella et al. [6] and Waldman et al. [7] both reported greater work without greater lactate concentrations following betaine supplementation. We propose two possible mechanistic explanations, either in isolation or combination, that may explain this paradox.

First, betaine ingestion enhances the methyl balance by transmethylating homocystine into methionine, which then is adenylated to form the universal methyl donor *s*-adenosylmethionine (SAM) [28]. SAM is then used to methylate guanidinoacetate to synthesize creatine [29]. If skeletal muscle phosphocreatine (PCr) concentrations are increased, adenosine triphosphate (ATP) synthesis may be shifted slightly away from glycolytic metabolism, and the resultant production of lactate, to PCr catabolism. Indeed, many studies have reported lower post-exercise lactate concentrations concomitant with increased anaerobic work following 5–7 days of creatine supplementation [30, 31–34]. While betaine administration has been shown to increase skeletal muscle PCr in live stock [35], to our knowledge, only one study has been conducted on this topic in humans. Del Favero et al. [14] reported 10 days of 2 g/day betaine supplementation did not increase skeletal muscle PCr concentration nor augment an increase in muscle PCr when combined with creatine monohydrate supplementation. As noted by Cholewa et al. [2], subjects in Del Favero et al. [14] were untrained and instructed not to exercise during the study period, and therefore minimized the demand for additional SAM to support endogenous creatine synthesis and skeletal muscle creatine uptake. In support of this hypothesis, skeletal muscle creatine concentrations are increased as a result of exercise [36], and exercise promotes greater muscle creatine accumulation when combined with creatine supplementation [37]. However, no mechanisms were determined in this study in regards to intramuscular creatine metabolism or endogenous creatine synthesis, so definite conclusions cannot be made.

Second, plasma lactate concentrations reflect the difference between lactate rate of appearance and rate of disposal, in which oxidation by the working tissue represents a significant source of lactate disposal [38]. Betaine protects cells against osmotic stress by redistributing water, which in turn leads to more effective biopolymer hydration and increased cytoplasmic osmolality [39]. In particular, physiological concentrations of betaine have been reported to defend citrate synthase, one of the rate limiting enzymes in the Krebs cycle, against thermo and urea denaturation [40]. Betaine has also been shown to upregulate cytochrome c oxidase activity [41], the terminal enzyme of the mitochondrial electron transport chain, and promote mitochondrial biogenesis [41–43]. We therefore propose that by enhancing mitochondrial respiration, betaine supplementation may have increased lactate oxidation, leading to the lower plasma concentrations observed despite significantly greater anaerobic work. Importantly, no assessment of mitochondrial biogenesis or function or oxidative energy metabolism was made in this study, so speculation still remains.

In the present study, short-term betaine supplementation significantly changed the indices of endocrine function in adolescent males. To the best of our knowledge, this was the first study to investigate the interaction between betaine supplementation and the acute steroid hormone response to RE. We observed that betaine administration for 14 days resulted in significant increases in plasma testosterone responses (10 ± 1.5 vs. 3.5 ± 1.6 ng.mL^−1^) after a session of high-intensity RE. There are three mechanisms that may explain the greater resting and exercise induced testosterone response with betaine supplementation.

First, there appears to be a relationship between the volume of work completed in a resistance training session and the testosterone response [44], and subjects completed significantly more repetitions in the both the bench press (10.2, 95% CI: 9.2, 11.2) and leg press (11.0, 95% CI: 8.9, 13.1) in the betaine condition over the course of 5 sets. Second, several studies have shown that endurance training can suppress basal testosterone levels and the testosterone response to exercise compared to controls [45]. These reductions in testosterone are hypothesized to be the result of increased hypothalamic, pituitary and/or testicular oxidative stress associated with the immune, thermal, and mechanical stressors of chronic aerobic exercise [46,47]. Betaine has been reported to ameliorate arsenic-induced testicular oxidative stress and plasma testosterone in mice by restoring testicular glutathione peroxidase, superdioxide dismutase, tissue catalase, and malondialdehyde [48]. While these results have not been replicated in humans, subjects in the present study were engaged in high intensity aerobic and interval exercise as part of their handball training, and had basal testosterone levels on the lower end of the normal range for 16-18 year olds [49]. We therefore hypothesize that betaine may have affected basal and exercise induced testosterone by restoring testicular function.

Finally, testosterone secretion involves the coordination of the hypothalamic pituitary adrenal testicular axis, whereby gonadotropin releasing hormone (GnRH) triggers the release of luteinizing hormone, leading to an increase in testosterone secretion. On the other hand, testosterone suppresses corticotrophin releasing hormone (CRH) and vice versa [50]. Whether betaine ingestion led to an increase in testosterone that suppressed cortisol or suppressed CRH to lead to greater testosterone concentrations in the present study is unknown. Nobari et al. [10] reported betaine supplementation raised basal testosterone but did not reduce cortisol following 14 weeks of supplementation in adolescent soccer players. Betaine ingestion in pigs [51] and carp [52] has been shown to increase betaine accumulation in the hypothalamus, leading to an increase in pituitary growth hormone releasing hormone and GH. Based on the aforementioned studies, we hypothesize that betaine may also accumulate in the hypothalamus of humans, leading to greater GnRH secretion, downstream increases in testosterone, and the suppression of CRH and reduced cortisol concentrations.

There are a few limitations in the present study that should be addressed. The rather small number of subjects was the main limitation of this study that raises interesting possibilities for future research, and the large effect sizes therefore should be viewed with caution. In addition, all participants were instructed to maintain their usual training and dietary routines throughout the study; however, their workload and nutrition on non-testing days was not standardized. Finally, the betaine product used in this study was purchased from a commercial retailer without an independent certificate of analysis, thus the possibility exists that the product label does not align with the actual contents.

Although there is a large volume of mechanistic data available from livestock and rodent studies, the results of this study have generated new hypotheses in need of research in humans. In particular, future studies are necessary to elucidate potential mechanisms responsible for the enhanced testosterone and metabolic responses to fatiguing resistance exercise with betaine supplementation.

## 5. Conclusions

Two weeks of betaine supplementation in adolescent males appeared to increase testosterone concentrations and decrease cortisol and lactate responses after a session of high-intensity RE. In addition, betaine intake improved the capability to perform more repetitions (at 80% 1RM) in the bench and leg press exercises, representing upper- and lower-body muscle endurance, respectively.

## Data Availability

The datasets used and/or analyzed during the present study are available from the corresponding author on reasonable request.

## Publication statement

This manuscript has not been published elsewhere and has not been submitted simultaneously for publication elsewhere.
